# Imagery of internal structure and destabilization features of active volcano by 3D high resolution airborne electromagnetism

**DOI:** 10.1038/s41598-019-54415-4

**Published:** 2019-12-04

**Authors:** Marc Dumont, Aline Peltier, Else Roblin, Pierre-Alexandre. Reninger, Stéphanie Barde-Cabusson, Anthony Finizola, Valérie Ferrazzini

**Affiliations:** 1 Sorbonne Université, CNRS, EPHE, UMR 7619 METIS, F-75005 Paris, France; 2 Université de Paris, Institut de physique du globe de Paris, CNRS, F-75005 Paris, France; 3 Université de La Réunion, Laboratoire Géosciences Réunion, F-97744 Saint Denis, France; 4 Observatoire Volcanologique du Piton de la Fournaise, Institut de physique du globe de Paris, F-97418 La Plaine des Cafres, France; 5 Bureau de Recherches Géologiques et Minières (BRGM), UMR 7327, BP 36009, F-45060 Orléans, France; 6 Hawai’i Institute of Geophysics and Planetology, School of Ocean and Earth Science and Technology, University of Hawai’i at Mānoa, Hawaii, United States of America

**Keywords:** Natural hazards, Solid Earth sciences

## Abstract

Present-day volcano imaging and monitoring relies primarily on ground surface and satellite remote sensing observations. The overall understanding of the volcanic edifice and its dynamics is thus limited by surface investigation, spatial resolution and penetration depth of the ground methods, but also by human and material resources, and harsh environments. Here, we show for the first time that an airborne electromagnetic survey provides a 3D global resistivity model of an active volcano. The high-resolution survey acquired at the *Piton de la Fournaise* volcano on *La Réunion* Island, Indian Ocean, shows unprecedented details of the internal structure of the edifice, highlighting the upwelling hydrothermal system below the craters, magma intrusion pathways and inherited faults. Together with surface monitoring, such airborne imagery have a high potential to better characterize volcano internal structure and magmatic processes, and therefore to better anticipate catastrophic events such as phreato-magmatic eruptions or volcano destabilizations.

## Introduction

Structures of active volcanoes result from many successive constructive and destructive processes that are interconnected^1–3^. Understanding their internal structure is a priority issue to monitor volcanic activity and anticipate catastrophic events such as phreatic to phreato-magmatic explosions, edifice collapses or flank destabilizations. Volcano edifices grow from both exogeneous and endogeneous processes through accumulation of lava flows, explosive deposits and magmatic intrusions. Successive magma intrusions and magma storages at depth generate accumulated deformations and mechanical stresses that weaken the volcano edifice stability, together with structures inherited from previous destructive events^1,4^. Water circulation also contributes to the overall complexity and instability of volcanoes. Heating of the water table by magma injections and accumulations at depth generates extensive hydrothermal systems underneath active volcanoes^5^. The induced circulation of acid hydrothermal fluids leads to intense leaching and alteration^6^. All these internal processes promote surface rupture^7^ and significant edifice spreading^8^ that can cause catastrophic flank destabilization^9^. Imaging the interior of active volcanoes is thus fundamental to determine their internal structure, the dynamic of their magmatic and hydrothermal systems, their potential points of failure and their associated volcano hazards such as phreatic/phreato-magmatic eruptions^10^, large-scale deformation^11,12^ and flank instabilities^2,13^.

**Figure 1:**
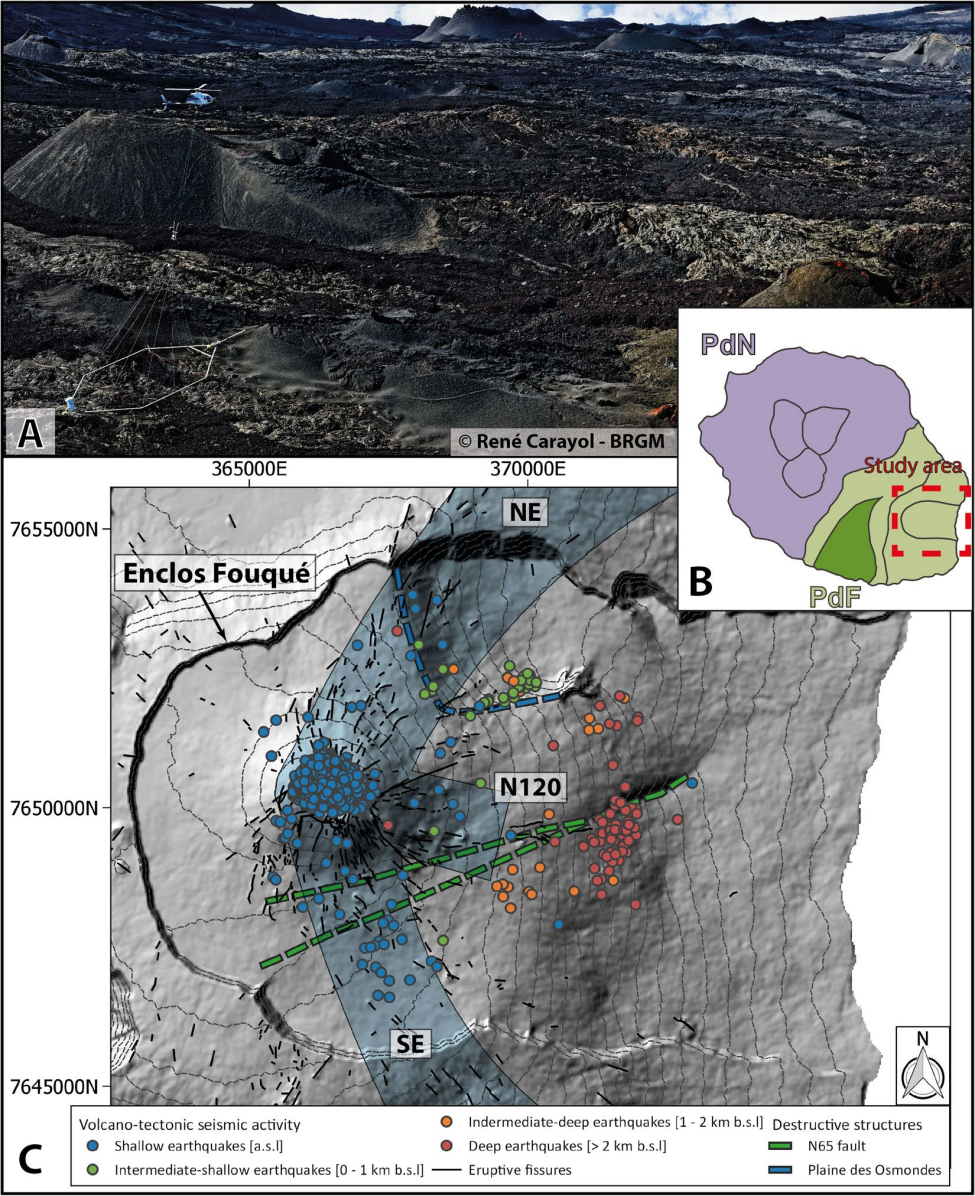
Presentation of the most active part of *Piton de la Fournaise*: the *Enclos Fouqué* caldera. (**A**) skyTEM device over the *Piton de la Fournaise* volcano illustrating the complexity of implementing ground based geophysical methods (© René Carayol and BRGM). (**B**) Map of *La Réunion* Island with the two volcanoes: PdN: *Piton des Neiges* - PdF: *Piton de la Fournaise*. The study area is delimited by a red dashed square. (**C**) Digital elevation model with the location of the recent volcano-tectonic seismic activity (colored circles), eruptive fissures visible in the field (in black solid line), the three rift-zones: NE, SE and N120 (Bachèlery, 1981), and the N65 (in green dashed lines) and *Plaine des Osmondes* (in blue dashed line) faults as defined by Michon and Saint-Ange (2008). Maps B and C have been generated with Illustrator CS6 and the open source QGIS 3.8.3, respectively. Coordinates in meters (WGS84, UTM 40S).

For decades, geophysical techniques have been developed and applied to study both the internal structure and the dynamics of active volcanoes. Among others, ground resistivity imagery techniques, such as 2D or 3D electrical resistivity tomography (ERT)^10,14,15^, magnetotellurics (MT) and transient electromagnetic soundings (TDEM)^16,17^, are renowned for being well-suited and reliable in such settings. While ERT oftenly provides high resolution imagery of the first hundred meters^18,19^, MT and TDEM provide a smoother image of the bulk resistivity up to few kilometres depth^20^. All these methods have been improved during the last decades to image resistivity variations in 3D^19,20^. However, ground acquisitions in steep and harsh environments, as it is the case on the *Piton de la Fournaise* volcano (Fig. 1A), often limit the spatial coverage and/or the resolution of the resulting 3D resistivity model. Depending on the objective, such ground based geophysical methods are able to provide either high-resolution imagery on small areas or low-resolution imagery on larger areas. This makes it difficult to assess a three dimensional and detailed view of the volcano internal structure.

**Figure 2:**
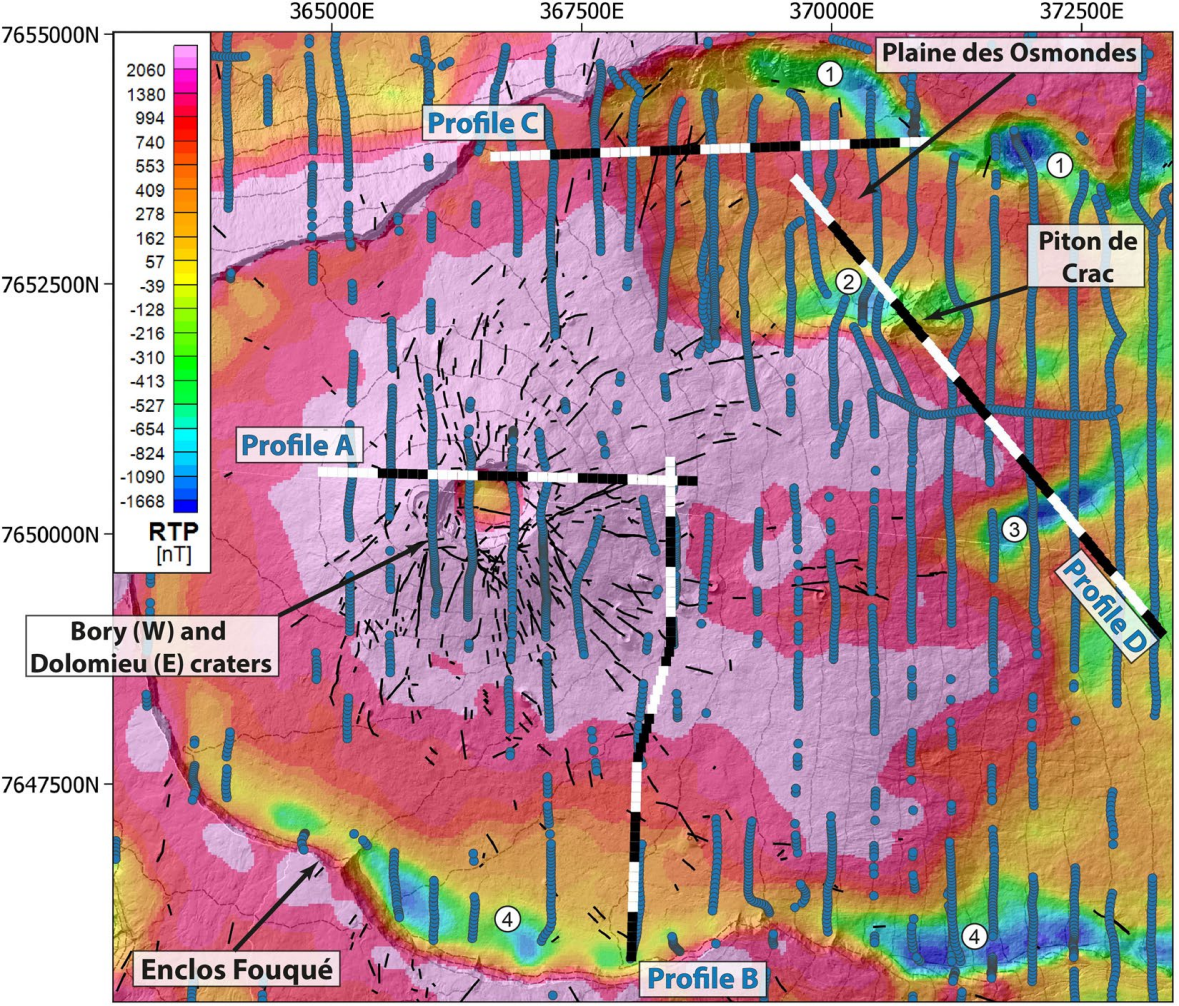
Location of the 3 655 inverted AEM soundings (blue dots) above the active part of the *Piton de la Fournaise* volcano (i.e. the *Enclos Fouqué* caldera), displayed on the magnetic anomaly map (reduced to the pole to lined-up anomalies with magnetic sources). The eruptive fissures visible in the field are denoted in black. The number refers to the negative magnetic anomalies discussed in the text. The 2D profiles (**A**–**D**) are marked with white/black lines (the color of the line changes each 500 m). The map has been created using open source QGIS 3.8.3. Coordinates in meters (WGS84, UTM 40S).

Here, we demonstrate the potential of airborne electromagnetic (AEM) surveys^21,22^ to image the resistivity contrasts within an active volcano such as *Piton de la Fournaise* on *La Réunion* Island (Indian Ocean; Fig. 1B) and its ability to provide high-resolution data over large areas and over shorter acquisition time than any other methods. For the past 40 years, this highly active, hot-spot, basaltic shield volcano has erupted every 8 months on average^23^. Its strong activity is at the origin of rapid changes in its morphology, both by endogeneous and exogeneous growth during effusive eruptions, and by destructions during periods of rare explosive eruptions (every 100–1000 years), collapses and flank landslides^8^. Most eruptions occur along one of the three rift-zones^24^ (NE, SE and N120; Fig. 1C), within the *Enclos Fouqué* caldera, that are about 3000–5500 years old^25^, and forms a U-shape structure opened to the east toward the sea. Surface eruptive fissures are fed by vertical dykes or horizontal sills originating from a shallow magma plumbing system^23,26^. This latter is composed of several reservoirs vertically distributed and connected, the shallowest being located 2–2.5 km below the surface within the volcano edifice^23,27,28^. This shallow magma network drives a well-developed hydrothermal system^29,30^ resulting in upwelling fluid circulations focused below the two summit craters (Dolomieu to the east and Bory to the west; see Fig. 2 for location) and the rift-zones^29,31^ (Fig. 1C).

The AEM survey were acquired over the *Piton de la Fournaise* volcano with a good acquisition coverage during this campaign (6 285 soundings over 150 km²). In spite of the high resistive nature of the medium in the near-surface, we successfully inverted 3 655 time domain electromagnetic decays (Fig. 2) after a data processing^32^ adapted to particularly resistive environments. Through this important undertaking, we obtain a 3D resistivity model of the internal part of the active volcano down to 1 kilometre depth. Our model shows on a wider spatial scale a much more detailed image of the structure than previous ground investigations. The resulting 3D resistivity model is compared to the magnetic data also acquired during the AEM survey, a self-potential (SP) map^29^ and the seismicity to validate imaged structures and investigate their potential link with the stability of the edifice. We focus our study on the three major features that can generate destabilization on volcanoes: (1) the hydrothermal system that can have link with volcano spreading^8^, future edifice collapses^10^ and phreato-magmatic explosions^10,33^, (2) dykes accumulations in depth providing an overview of the preferential magma pathways, and (3) faults inherited from previous destabilizations, susceptible to create future destabilization planes.

## Results

### 3D geometry of the hydrothermal system

**Figure 3:**
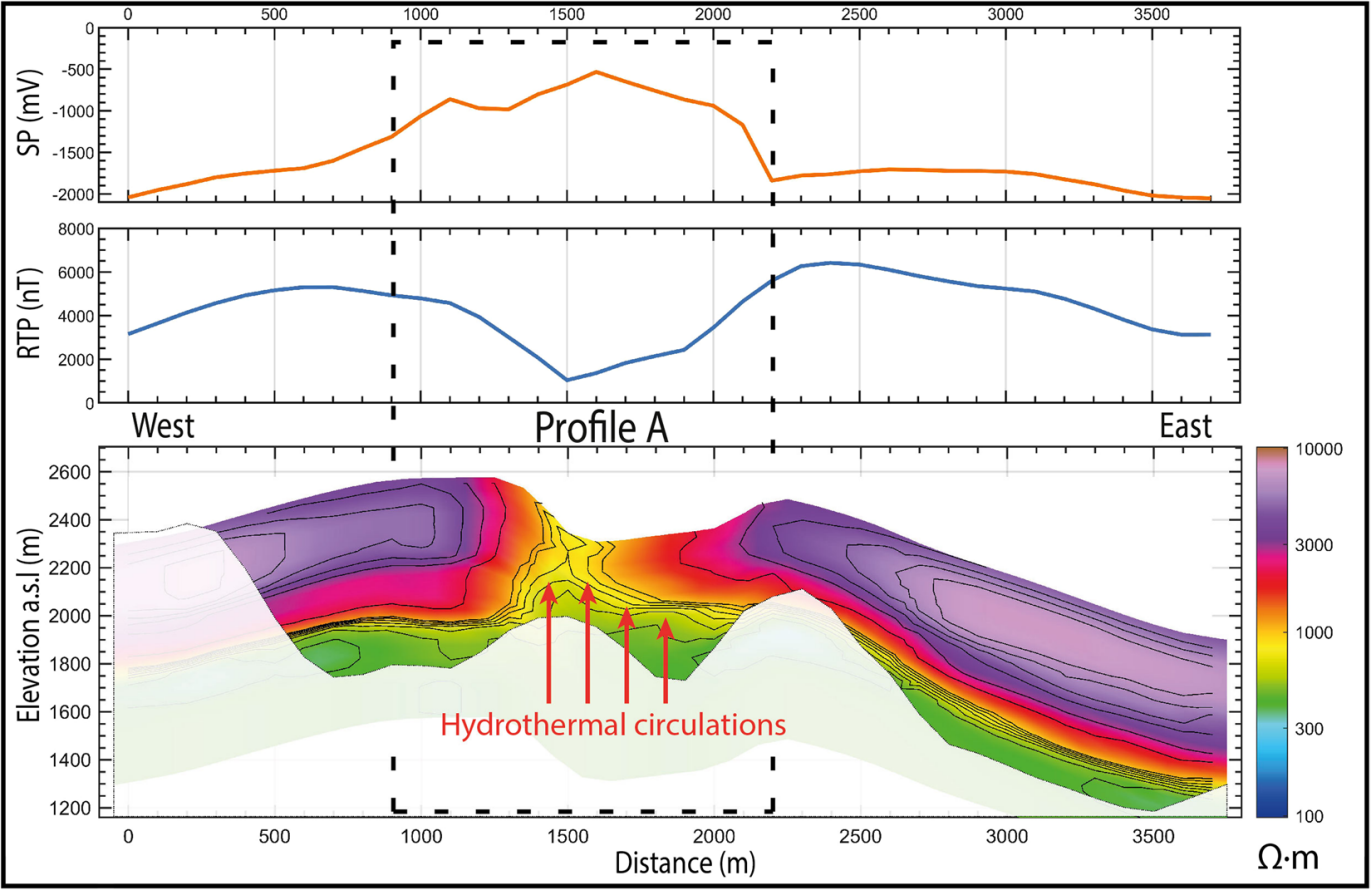
Imagery of the terminal cone of the *Piton de la Fournaise* volcano. 2D profile A of self-potential values (top panel), RTP magnetic anomaly (middle panel) and resistivity (bottom panel). Areas below the depth of investigation are blanked. The black dashed rectangle delineates the hydrothermal system imaged with SP positive anomaly. The location of profile A is shown on Fig. 2.

**Figure 4:**
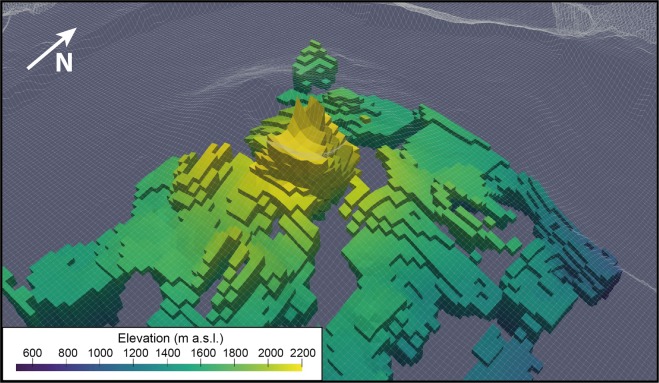
3D extraction of the conductive body from the AEM resistivity model. The model is shown as a function of the elevation of each cells.

**Figure 5:**
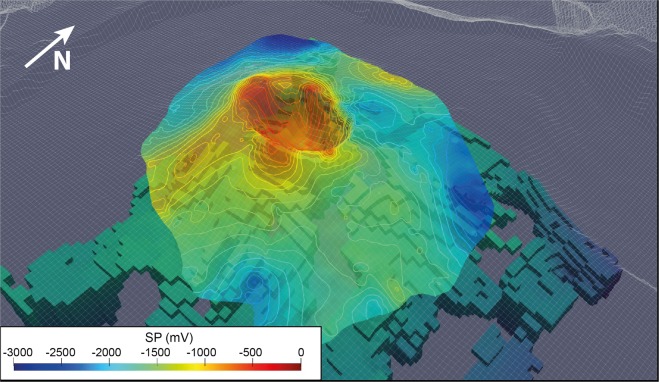
3D extraction of the conductive body from the AEM resistivity model. The model is shown as a function of the elevation of each cells overlayed with the self-potential map^29^.

**Figure 6:**
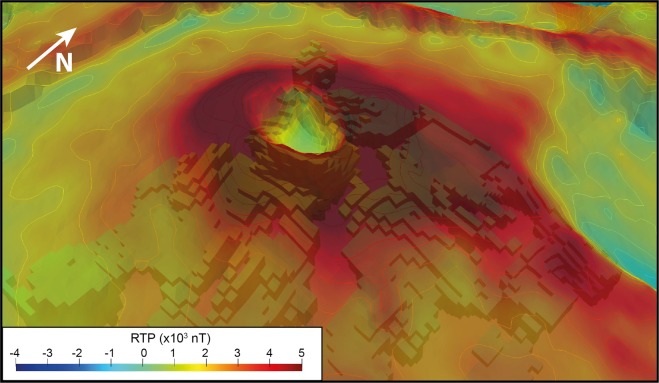
3D extraction of the conductive body from the AEM resistivity model. The model is shown as a function of the elevation of each cells overlayed with the magnetic RTP map.

As the bulk resistivity is notably controlled by clay content, water saturation and mineralization^34^, we first explore the potential of AEM survey to characterize the hydrothermal system. Figure 3 shows 2D resistivity, magnetic anomaly and SP profiles across the Dolomieu crater. Over both the western and eastern flanks of the volcano, the AEM method images a 500 m thick shallow resistive layer (>2 000 Ω.m), lying on top of a more conductive layer (<1 200 Ω.m). Those observations are fully consistent with a previously acquired 3800 m long electrical resistivity tomography (ERT) profile on the northern flank of the terminal cone^35^, but with a better coverage beneath the flanks and the crater. AEM imagery further evidences a conductive body rising to the surface below the Dolomieu crater, associated with a magnetic anomaly decreasing from 5 000 nT at the crater rim to 2 000 nT at its center (Fig. 3, middle panel). A SP survey^29^ images a positive SP anomaly from −2 000 mV to −500 mV over the same area. To better visualize the lateral variations of the conductive body (<1 200 Ω.m), we extract its 3D geometry from the resistivity model (Fig. 4). Below the Dolomieu crater, the conductive body forms a circular funnel, consistent with the circular inverse magnetic and positive SP anomalies observed below the same area (Figs. 5 and 6). Outside the crater, on the flanks, the deep conductive body follows the topography as imaged in the 2D profile (Fig. 3, bottom panel). Another view of the conductive body is shown in Supplementary S1.

Previous SP surveys in the vicinity of the Dolomieu crater mapped positive SP anomalies consistent with upwelling hydrothermal water and gas flows below the craters, especially to the west where hydrothermal fluids circulate within previously defined structural limits^29,36^. The presence of such warm mineralized groundwater and gas lowers the bulk resistivity, and induces hydrothermal alteration, that further decreases resistivity and magnetization of volcanic layers^17,37^. The presence of the circular conductive body, magnetic and SP anomalies below the crater leads to the conclusion that the funnel below the crater corresponds to the shallow hydrothermal system of *Piton de la Fournaise* (Fig. 4). On the flank of the volcano, the absence of SP and magnetic anomaly points to the absence of shallow hydrothermal system. Thus, the deep conductor likely corresponds to old weathered volcanic rocks previously imaged through electromagnetic methods^38^. In the 2D profile, the resistivity of old weathered lava flows, called the basement (200–600 Ω.m), is lower than that of the shallow hydrothermal system (500–1 200 Ω.m; Fig. 3, bottom panel). The first one consists of old highly weathered/altered lava flows, whereas the second one could correspond to recent lava flows partially saturated in hydrothermal water and gas. The higher resistivity of this latter in confront to the basement could be explained both by a weaker alteration induced by the hydrothermal fluids than a higher quantity of vacuum (resistive body) induced by previous caldera collapses.

The 3D resistivity model shows that hydrothermal circulations are mainly localized beneath the craters. The intensity of hydrothermal upwelling varies from the western to the eastern part^29^, the two parts being bounded by structural limits. To the east of the crater, higher bulk resistivity and magnetism values are associated with less altered rocks, while to the west lower resistivity and magnetism values reflect a more active hydrothermal system (Fig. 3). The resulting leaching and alteration processes likely weaken volcanic rocks, preferentially to the west. The evolution of the rock mechanical properties could generate a slow spreading of the terminal cone, which, in the future, might activate inherited faults delineating the western and eastern parts of the Dolomieu crater^29,39^. Such spreading could explain part of the continuous displacement of the eastern flank of the volcano towards the sea (about 1–2 cm per year)^39–41^. Finally, the presence of a developed hydrothermal system at shallow depth accentuates the risk of phreato-magmatic eruptions at *Piton de la Fournaise* in case of rapid rise of magma to the surface or a lateral drainage of magma^30^, two conditions that may induce magma- water interactions and therefore explosive activities.

### Magma injection pathways within rift-zones

The shallow hydrothermal system is primarily located beneath the craters. Another significant source of destabilization arises from the accumulation of magma injections mainly within the rift zones that further weakens the edifice^1^. Dykes can be located either by eruptive fissures at the surface, or by inversion of ground deformation dataset^12,42,43^ at depth. The latter method provides accurate estimations only for one or a few magmatic intrusions. We thus analyze the opportunity offered by the AEM method to obtain a comprehensive view of magma preferential pathways.

**Figure 7:**
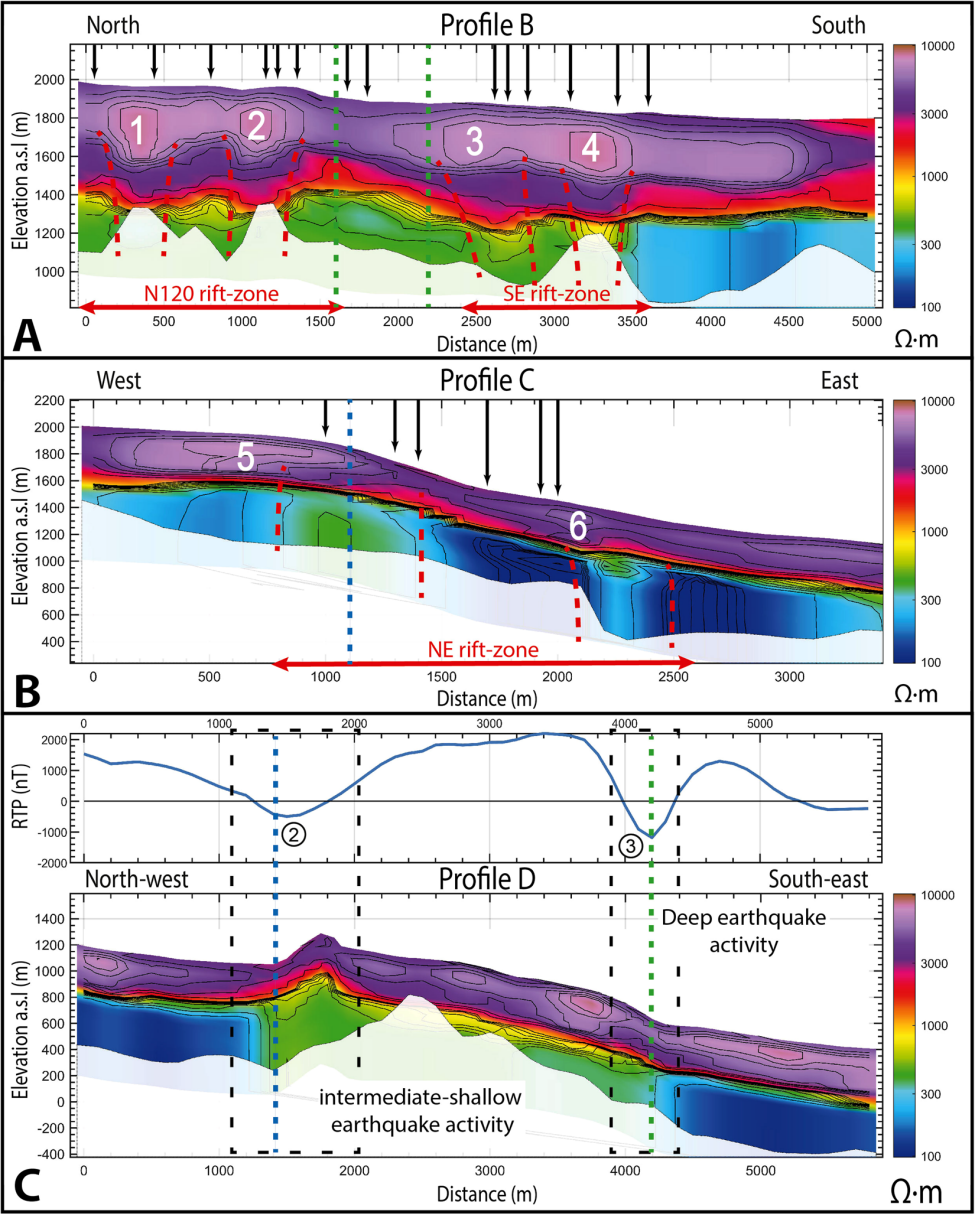
(**A**) 2D resistivity profile B, (**B**) 2D resistivity profile C and (**C**) 2D resistivity profile D. Areas below the depth of investigation are blanked. As no significant magnetic variations are present on profile B and C, the magnetic anomaly (reduced to the pole to lined-up anomalies with magnetic sources) is only shown for profile D. The magnetic anomaly 2 and 3 are appointed in the profile D. Each resistive core is numbered: 1–2 for the N120 rift-zone, 3–4 for the SE rift-zone, and 5–6 for the NE rift-zone. The location of eruptive fissures is indicated by black arrows on the profile. The N65 and Plaine des Osmondes inherited faults are denoted by dashed green and blue line, respectively. The vertical resistivity structures interpreted as magma injection pathways are outlined with dashed red lines. In profile D, the two black dashed rectangles represent the lateral extension of earthquake activity. See Fig. 2 for the location of each profile and the magnetic map.

Profile B crosses the N120 and SE rift-zones from North to South (Fig. 2). The near-surface is characterized by a 600 m thick resistive body overlaying a conductive body (Fig. 7A). Above the two rift-zones, four distinct highly resistive cores (>8 000 Ω.m) are visible at the near-surface (1–2 and 3–4 on Fig. 7A for the N120 and the SE rift-zones, respectively). The base of these cores forms a root extending to 50 to 100 m depth, displaying higher resistivity than the underlying substratum. This alignment creates vertical resistive structures (dashed red lines on Fig. 7A). On the profile C crossing the third rift-zone (NE), we image the same highly resistive geo-electrical structure (400 meters thick) on top of a conductive body, and two vertical resistive structures within the rift-zone (Fig. 7B). The profile C crosses the *Plaine des Osmondes* fault inherited from a previous destructive event^8^, creating a steep topographic variation. The substratum roots, resistive cores and surface eruptive fissures are shifted (Fig. 7B). In the upper part, eruptive fissure and the root are located at the eastern limit of the resistive core 5, nearby the topographic depression. In the lower part, while eruptive fissures are located at the west, the roots are located at the east of the resistive core 6. The presence of structures inherited from previous destructive events^8^ may impact the distribution of magma injection pathways near the fault.

Our AEM global resistivity model is in good agreement with the geo-electrical models deduced from previous geophysical studies^17,20^, validating the global trend of our more detailed imagery. At the near-surface, the resistive layer - several hundred meters thick - corresponds to recent lava flows. Below, the conductive layer has been interpreted as old weathered lava flows from previous eruptive phases^38^. Our 3D imaging shows specific patterns of resistivity variations within the rift-zones. As demonstrated at Garrotxa volcano, magma pathways are imaged by vertical resistive structures^44^. At *Piton de la Fournaise*, eruptive fissures at the surface are lined-up with shallow dense resistive cores and more resistive layers at depth (Fig. 7A,B). This vertical structure consists of magma injections crossing old weathered substratum at depth.

In the area of *Plaine des Osmondes*, the topographic depression created before the Enclos Fouqué caldera event^8,25^ controls the distribution of recent lava flows, with thicker accumulation at the top and the bottom of the slope (Fig. 7B). At depth, the increase in the substratum resistivity is likely a sign of the presence of dykes swarms. Compared with profile B (Fig. 7A), resistivity contrasts between the substratum and magmatic intrusion are more diffuse due to a lower volcanic activity that is mostly focused on the terminal cone^23^. Vertically crossing the substratum, the two magmatic pathways bend at 300 meters depth reaching the surface nearby the *Plaine des Osmondes* limit which should ease the circulation of magma.

All these results demonstrate the ability of the AEM method to image preferential magma injection pathways through the increase of the bulk resistivity. This result confirms that rift-zones are preferred areas for magma upwelling and provides a comprehensive view of their location and geometry. The complex structure of lava flows and magma injections controls future paths taken by the magma before reaching the surface^45^. Depending on the stress distribution, magma transit may be facilitated by extensive stress or stopped by compressive stress so that new magma injections reach the surface at the boundary of the resistive cores (see black arrows on Fig. 7A,B).

### Inherited faults from past destabilizations

As shown at *Plaine des Osmondes* (Fig. 7B), previous landslides and destructive events could significantly impact the magma injection pathways and therefore the volcanic activity. Thus, we explore the causal link between faults evidenced at the surface, their extension in depth and their possible impact on the volcano stability. The magnetic map from the AEM survey provides information on the main structures of the active volcano (Fig. 2). The terminal cone, where 97% of the recent eruptions occurred, consists of a thick pile of recent lava flows^30,39^ characterized by highly positive magnetic anomaly (>600 nT - Fig. 2). Downhill, the recent lava flow stack is thinner, resulting in a weaker average magnetic response. The eastern flank, facing the ocean, is characterized by a slightly positive to negative magnetic signature (between 200 to −500 nT). Between these two poles (terminal cone and downhill), the bulk magnetic signature decreases smoothly with the slope. Four highly negative anomalies in the middle of the eastern slope highlight the presence of hidden structures (see location on Fig. 2). Anomalies 1 and 4, at the top and the bottom of the northern and southern *Enclos Fouqué* caldera limits, respectively, are linked to old lava flows with negative magnetization close to the surface. Two other negative anomalies (numbers 2 and 3 on Fig. 2) are visible nearby *Piton de Crac* (remnant part of the *Piton de la Fournaise* scarp isolated by collapses and erosion^8,24^) and in the eastern part of the *Enclos Fouqué* caldera respectively. Anomaly 2 is located above a cluster of intermediate-earthquakes (between 0 and 2 km below sea level). Anomaly 3 corresponds to a topographic anomaly at the end of the N65 faults^8^, which is associated with the *Enclos Fouqué* caldera collapse formation^25^, above a cluster of deep earthquakes (>2 km below sea level). Resistivity profile D along the eastern flank crosses the negative anomalies 2 and 3 (Fig. 7C). It shows a highly resistive layer in near-surface (>2 000 Ω.m) all along the profile and three distinct parts in depth: i.e. a middle part below a topographical high - displaying medium resistivity (400–800 Ω.m) - well delimitated by two sharp vertical limits from the surrounding areas - displaying lower resistivity (100–200 Ω.m). Each deep and sharp limit coincides with a strong negative magnetic anomaly and volcano-tectonic seismic activity in depth.

*Piton de la Fournaise* destabilization significantly scarred the morphology of the volcano. Three consecutive collapses created the *Enclos Fouqué* caldera and the *Plaine des Osmondes* area^8^. Our AEM resistivity model highlights deep sharp structures below the topographic anomalies (notably the N65 and *Plaine des Osmondes* faults). We thus demonstrate the continuity between the surface topographic clues and our new imaged vertical structures at depth. Clusters of earthquakes are present below these faults and their vertical prolongation at depth evidenced by resistivity (Figs. 2 and 7C) suggests a continuity of both the *Plaine des Osmondes* and the N65 faults down to at least 1 km and 2 km b.s.l, respectively. As discussed above, the central cone is spreading slowly towards the sea leading to significant stress on the *Plaine des Osmondes* and N65 major faults. Together, the mechanical pressure from the spreading motion with magma circulations applied on inherited structures could lead in the future to fault re-activation and catastrophic destructive events. The localization and geometry of these inherited structures provided by AEM imagery will enhance our understanding, the set-up and interpretation of volcano monitoring and management of incoming destructive events.

## Discussion

Overcoming some AEM limitations in highly resistive setting, we obtain for the first time a 3D high resolution imagery of the internal structure of a large active volcano. Confronted with geological and other geophysical results, one AEM survey allows us to define (1) the 3D geometry of the shallow hydrothermal system upwelling below the craters, (2) the location and vertical pathways of the dense magma intrusion system along the three rift-zones, and (3) the extension of deep volcano-tectonic structures from surface lineaments to deep earthquake activity below the eastern mobile and unstable flank. The airborne dataset improves our understanding of the *Piton de la Fournaise* geological history and future possible evolution by imaging zones of weakness resulting from different volcanic processes and by supporting and validating previous hypotheses. Its ability to detect over large area the three major destabilization features, together with geological observations and/or geophysical measurements, provides critical information to understand their interconnection. We present a reliable geometry of the hydrothermal system, highlighting its presence near the summit and its possible role in the continuous eastern flank motion. We define six main magma injection pathways within the rift-zone, improving our understanding of magma pathways near the surface and eruptive dynamism. Finally, we further demonstrate that topographic scars from previous destructive events correspond to deep fault plans where volcano deformation accumulate mechanical stress which can be reactivated in future volcano destabilizations. In the future, the *Piton de la Fournaise* surveillance program will integrate weakness areas identified in this study to improve the efficiency of volcano monitoring. The integration of airborne results into realistic and detailed 3-D model of the edifice will also help to interpret the volcano dynamism and instabilities.

The knowledge of the internal structure of active volcanoes is a critical step to understand the eruptive and destructive events that shaped the edifice in order to better anticipate the next events. In such a resistive environment, the quality of AEM data is limited and requires fine processing methods. Future developments should be dedicated to recover more AEM soundings, improving thus both the coverage and the resolution of the method. Our work demonstrates the ability of AEM to image the major structures controlling the shallow volcano evolution such as the hydrothermal system, the magma injections and the inherited structures. The AEM method shows its high potential to image the inner structure of volcanoes with large depth of investigation (up to 1 km in this environment), high resolution (up to 30 m along flight lines spaced 200/400 m) and high coverage (hundreds square kilometres in few days), and an average density measurements up to 25–42 soundings/km². Irrespective of the volcano type and dynamism, AEM makes possible to locate areas of weakness on decametric scales and thus defines zones to be monitored in the future to mitigate the associated risks.

## Methods

In 2014, an airborne time domain electromagnetic survey was conducted over *La Réunion* Island^22^ (SkyTEM system^21^). This method uses transmitter loops to induce electrical currents in the ground and measure the resulting magnetic field. The SkyTEM system emits two different magnetic moment: (i) a low moment (3 100 A.m^2^–325 Hz) for near-surface resolution, and (ii) a high moment (160 000 A.m^2^–25 Hz) for depth investigation. During the acquisition, the signal-to-noise ratio is improved stacking numerous measurements together^46^. After the survey, electromagnetic noises (natural and anthropical) are removed applying dedicated filters^32^. First, a time threshold is defined in function of the instrumental characteristics and the noise level. Then, the capacitive filter will be applied within this time threshold culling data outside the two curves. Second, the electromagnetic decays are averaged using a sliding trapezoidal windows preserving a good resolution of the shallow depth, while improving the signal-to-noise ratio for imaging greater depths. Finally, the first filter is reapplied on the average decays. More information on the processing scheme is provided in the Supplementary S2. At the end, the entire dataset is manually edited to remove the remaining noises (e.g. galvanic noise). Each remaining EM data is then inverted into a 1D resistivity model using the quasi-3D Spatially Constrained Inversion scheme^47^. Each 1D model is defined by several layers defined by a thickness and a resistivity. For each obtained 1D model, an analysis of the inversion sensitivity provides an estimation of the depth of investigation (DOI)^48^. Below the DOI, the resistivity is blanked in the 2D and 3D figures (Fig. 7 - Supplementary S3). In order to obtain a 3D resistivity model, 1D vertical soundings are interpolated in two steps. First, for each layer, the resistivity value and thickness at each 1D model are interpolated (resistivity maps are presented in Supplementary S4). Second, the 2D maps are used to build a 3D irregular grid of resistivity called here 3D resistivity model^49^.

The initial processing has first been applied, at the island scale, to the whole AEM dataset. More than 80% of the EM decays acquired over *Piton de la Fournaise* were rejected because of the very low signal-to-noise ratio of the data in this area. First, as the substratum gets more resistive, the electromagnetic decays gets more and more steep. Indeed, in this area the substratum gets very resistive and the electromagnetic decays reached very rapidly the noise level. The initial processing was not fined tuned for this specific environment. It is therefore not surprising to obtain such bad results.

Given the very low signal-to-noise ratio of the studied area, we decided to reprocess the dataset with more suited parameters^50^. We only used the high moment decays and applied a wider stacking (the characteristics of the new filters are specified in Supplementary S1). Thanks to this reprocessing, usable AEM soundings over the volcano increased from 1 172 to 3 655 out of 6 285 measurements (location and DOI of AEM soundings are shown in Supplementary S3). A quasi-3D spatially constraint inversion has then been achieved to provide a five layers resistivity model. These first results provide attractive results in both volcanology and geophysics domains.

## Supplementary information

Supplementary information
