# Supplementary material for: Imagery of internal structure and destabilization features of active volcano by 3D high resolution airborne electromagnetism
Source: Sci Rep. 2019 Dec 4;9:18280. doi: 10.1038/s41598-019-54415-4 (PMC6892846; doi:10.1038/s41598-019-54415-4)
Supplement: Supplementary file 1 — Supplementary information [file 41598_2019_54415_MOESM1_ESM.docx]

Imagery of internal structure and destabilization features of active volcano by 3D high resolution airborne electromagnetism

**Authors:** Dumont, M., Peltier, A., Roblin, E., Reninger, P.-A., Barde-Cabusson S., Finizola, A., Ferrazzini, V.

**Supplementary – S1:** 3D views of the AEM conductive body of the *Piton de la Fournaise* terminal cone.


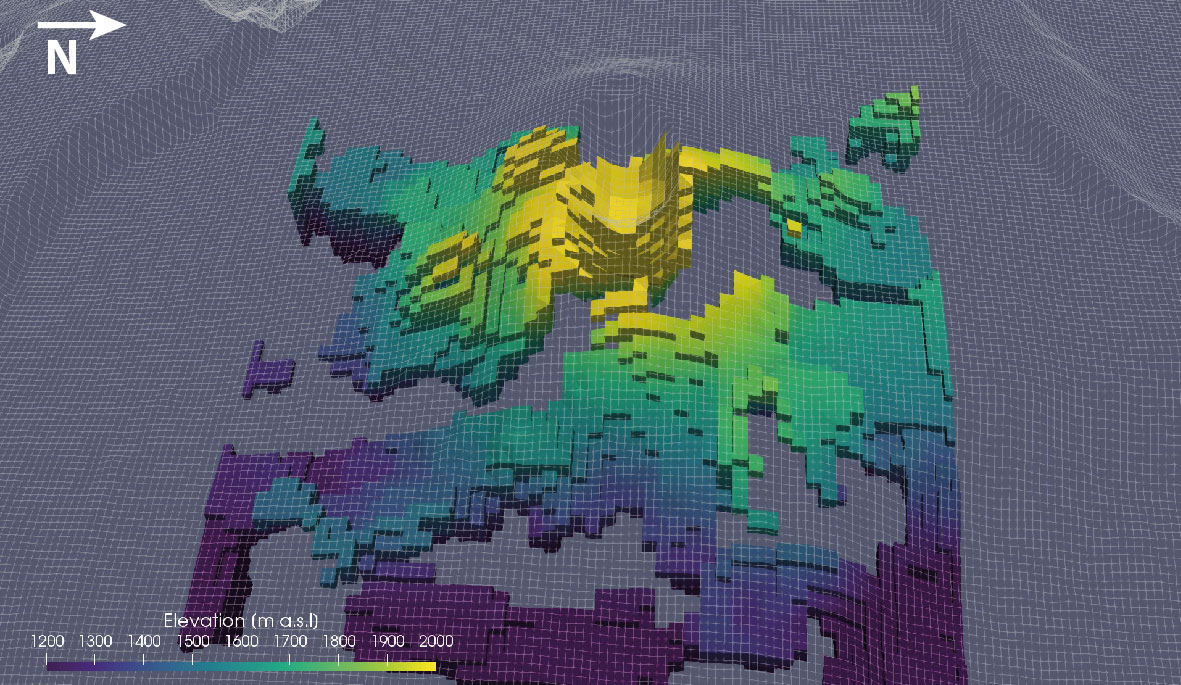


S1a - 3D extraction of the conductive body from the AEM resistivity model. The model is shown as a function of the elevation of each cells.

**
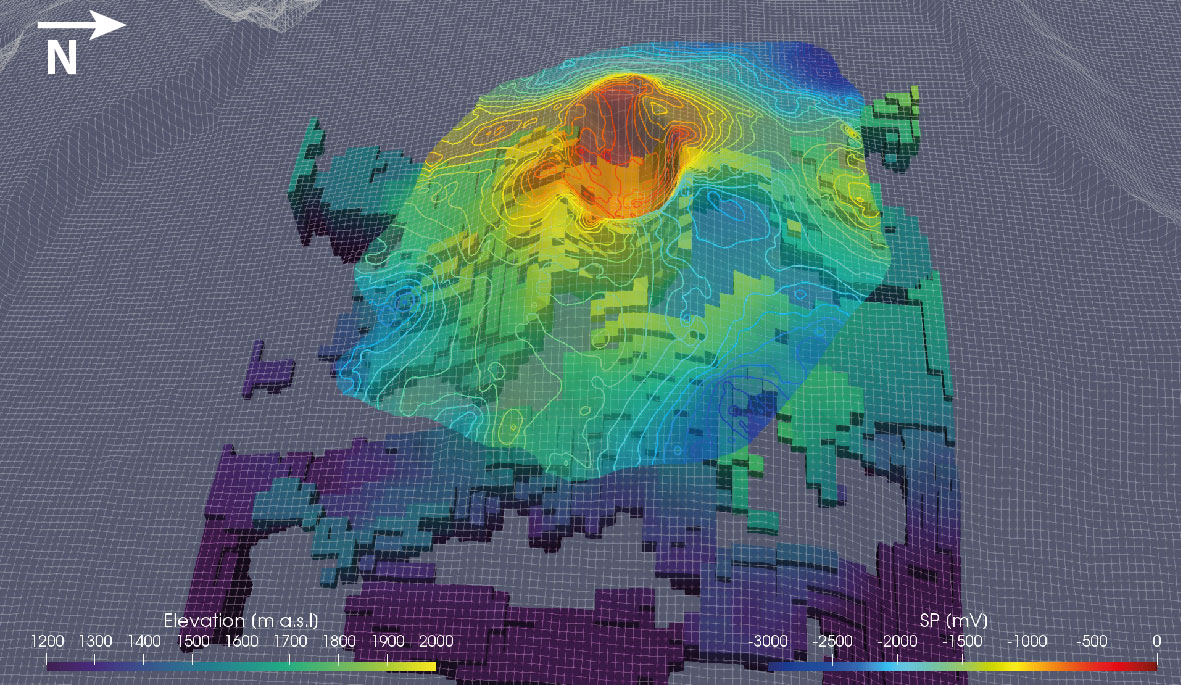
**

S1b - 3D extraction of the conductive body from the AEM resistivity model. The model is shown as a function of the elevation of each cells overlayed with the self-potential map.

**
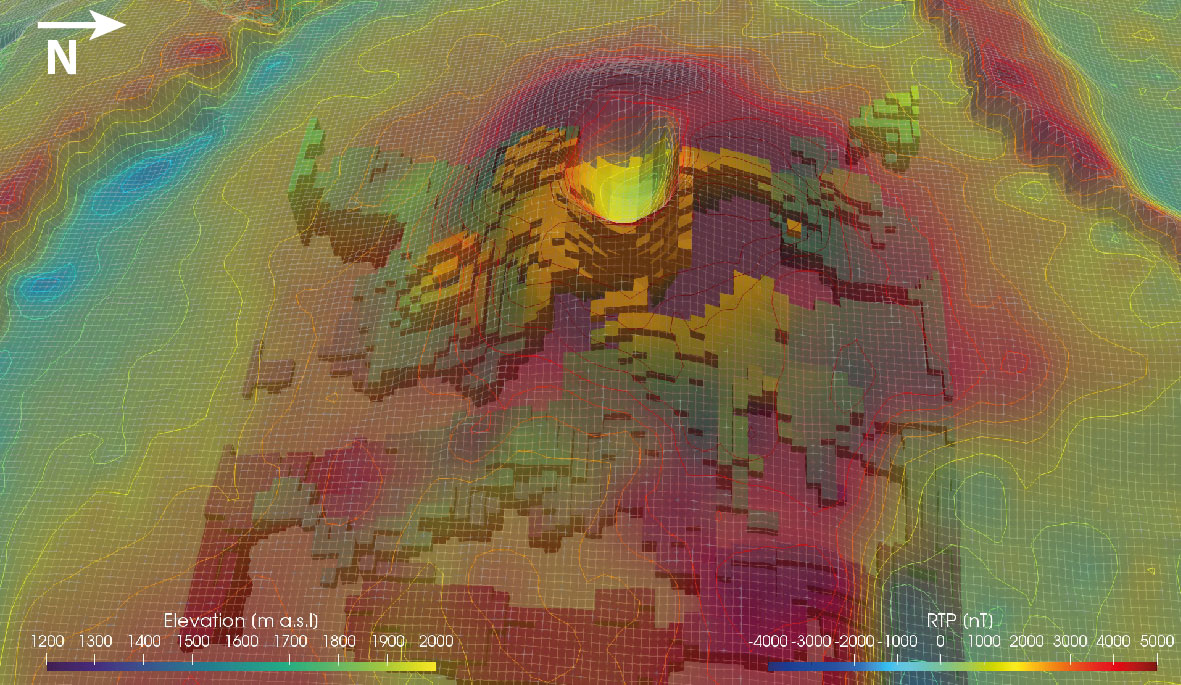
**

S1c - 3D extraction of the conductive body from the AEM resistivity model. The model is shown as a function of the elevation of each cells overlayed with the magnetic RTP map.

**Supplementary – S2: AEM data processing**

This supplementary presents the parameters and the processing of both processing schemes are specified in S2a and S2b. To understand the difference of both processing schemes, electromagnetic decays along flight lines and in function of time are presented in figure S2c.

|  | Initial processing scheme | New processing scheme |
| --- | --- | --- |
| **Time filter** |  |  |
| Starting time | 1.5∙10^-4^ | 1.5∙10^-4^ |
| Noise level at 1 ms (V/m²) | 3∙10^-7^ | 3∙10^-7^ |
| Noise level slope | -0.7 | -0.7 |
| **Capacitive slope filter** |  |  |
| slope start (s) | 3∙10^-7^ | 3∙10^-7^ |
| min slope | -0.6 | -0.6 |
| max slope | 0.6 | 0.6 |
| **Trapezoidal average windows** |  |  |
| T1 (at 10^-4^s) width (s) | 4 | 20 |
| T2 (at 10^-5^s) width (s) | 6 | 40 |
| T3 (at 10^-6^s) width (s) | 14 | 80 |
| **Average slope filter** |  |  |
| Filter start (s) | 6∙10^-4^ | 2∙10^-4^ |
| min slope | -0.5 | -0.5 |
| max slope | 0.5 | 0.5 |
| S2a – Characteristics of the initial and new processing scheme applied on AEM data. A description of each filter is presented in s2b. | | |


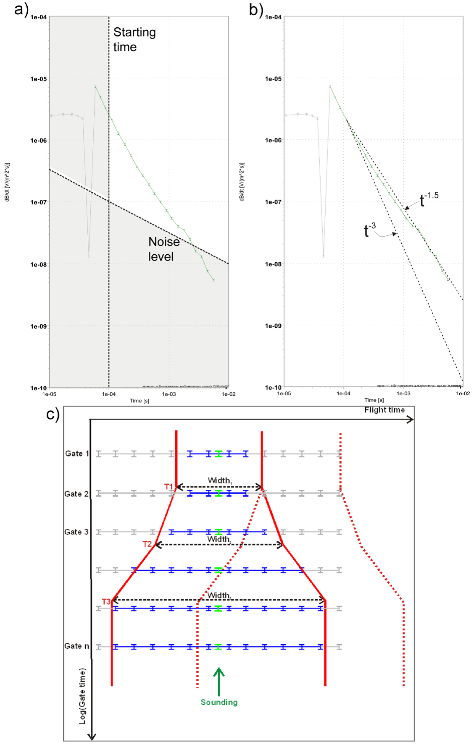


S2b – Sketch of the four filters applied on raw AEM data. (a) the time filter define a the time threshold of the electromagnetic decay. It is define by a starting time and a noise level. (b) capacitive and average slope filter reject data falling outside the area defined by the two lines. The two lines are defined by a starting time and a slope precised in S2a table. (c) the trapezoidal average windows stacks electromagnetic decays along flight lines. Tight at early time, the filter becomes wider with time since the signal to noise ratio increase and the resolution decrease. This figure has been modified from Auken et al. (2009).

References:

Auken, E. et al. An integrated processing scheme for high-resolution airborne electromagnetic surveys, the SkyTEM system. Explor. Geophys. **40**, 184–192 (2009).


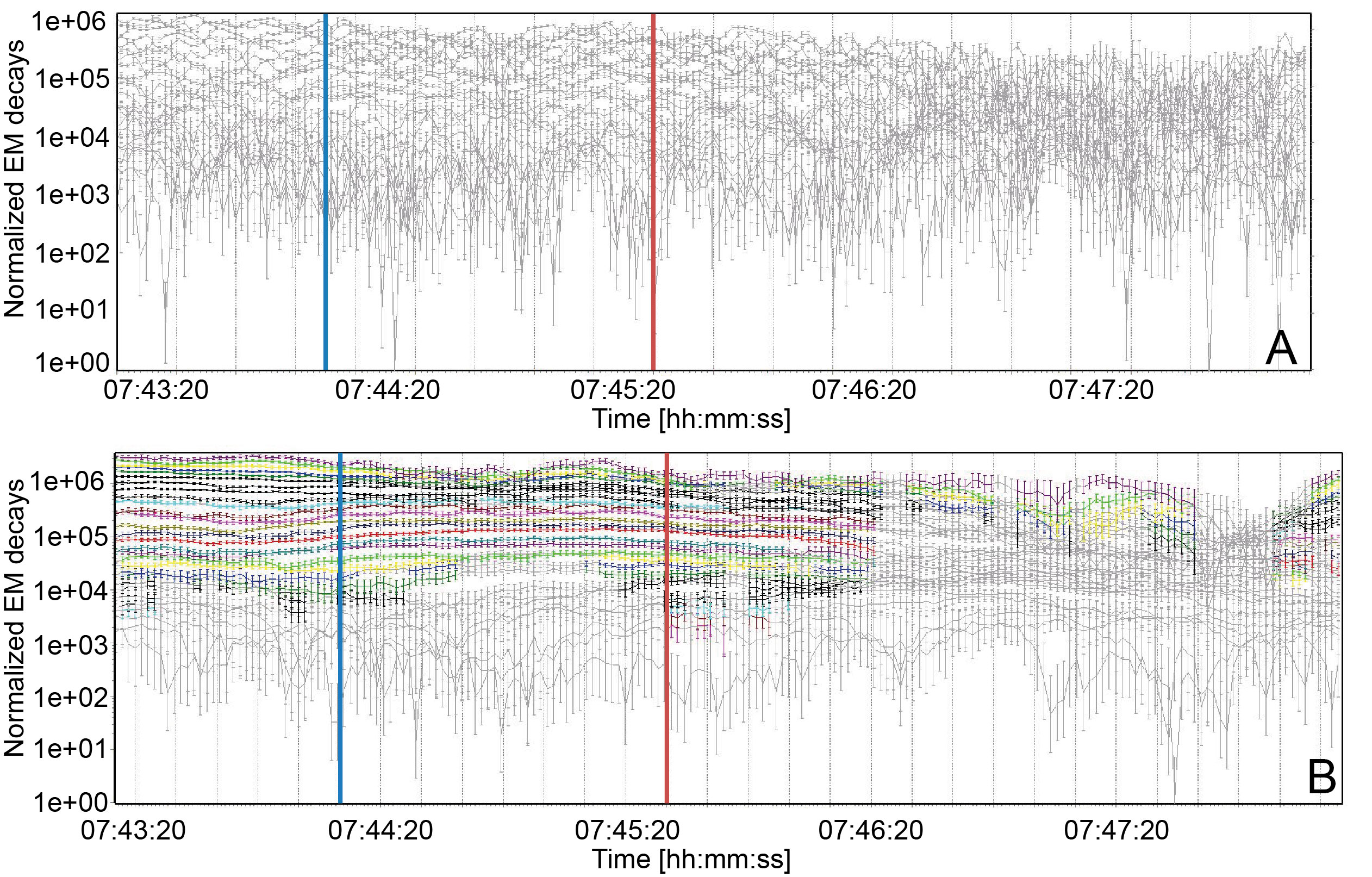
S2c – electromagnetic decays, along flight line. A] Data after the standard initial processing scheme. B] Data after the new processing scheme. The blue and orange lines specified point the electromagnetic decays displayed in S2d.


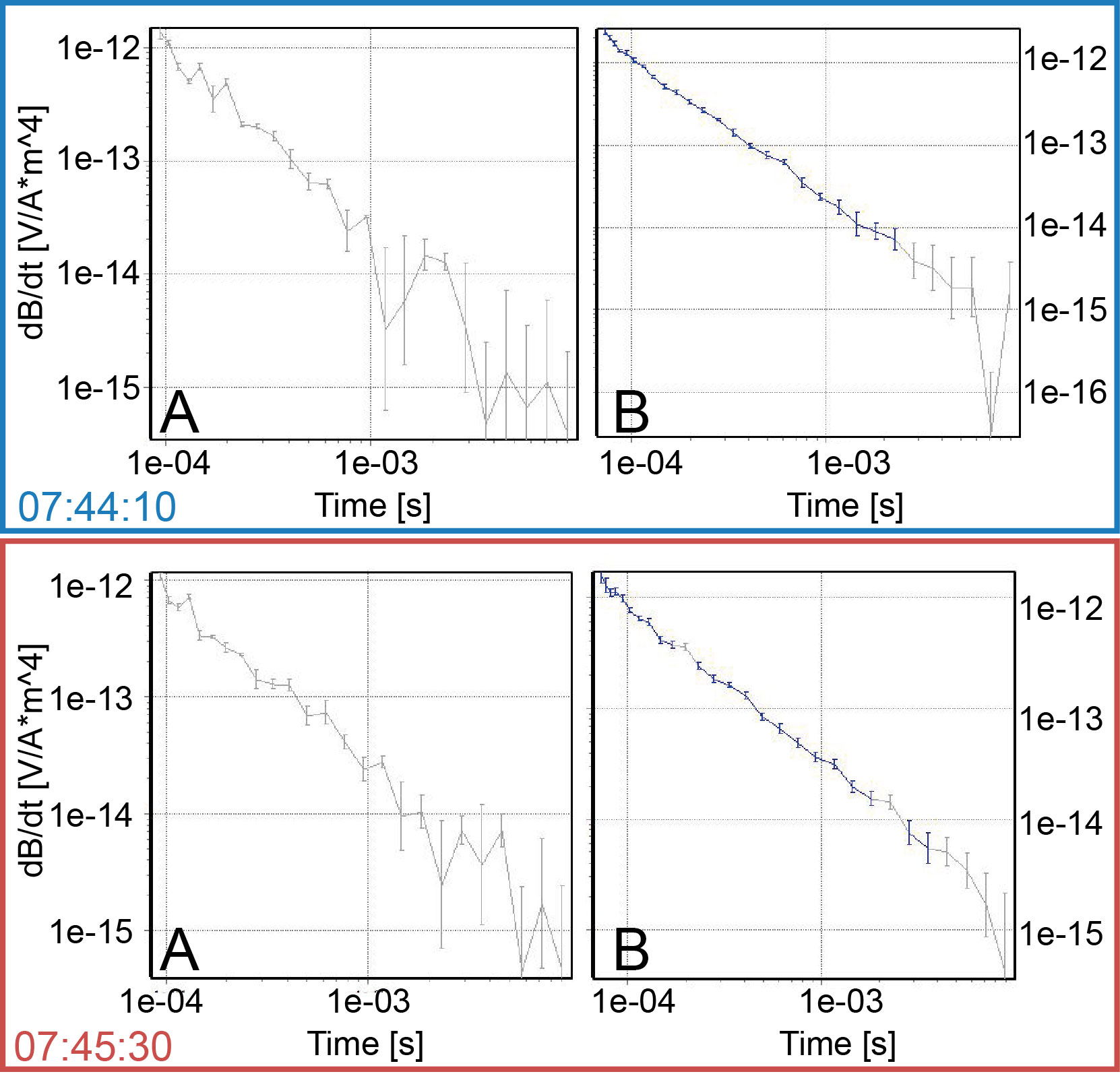

S2c – two electromagnetic decays in function of the time. The blue and orange squares correspond to the colored lines in the flight lines S2d.
A] Data after the standard initial processing scheme. B] Data after the new processing scheme.

**Supplementary – S3:** Airborne electromagnetic survey over the *Piton de la Fournaise* volcano.

| 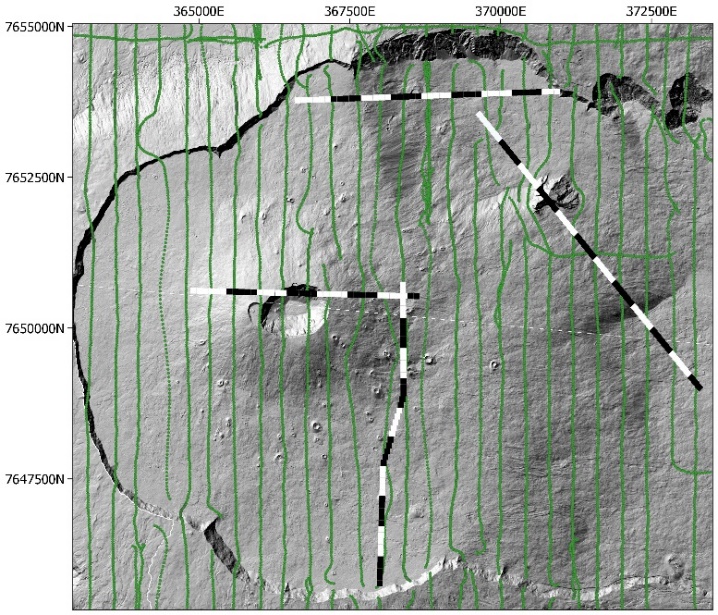  S2a - Location of the 6 285 Time Domain Electromagnetic measurements (green dots) acquired during the AEM survey in 2014. | 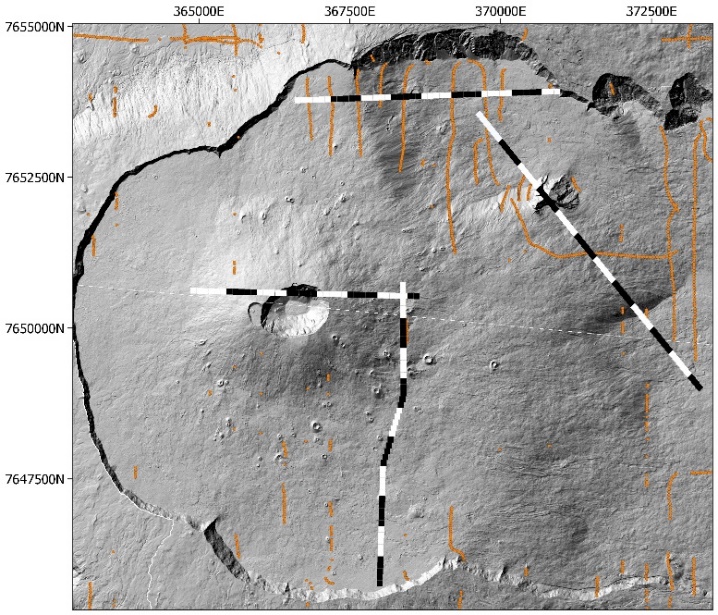  S2b - Location of the 1 172 inverted soundings (orange dots) after the standard processing scheme. |
| --- | --- |
| 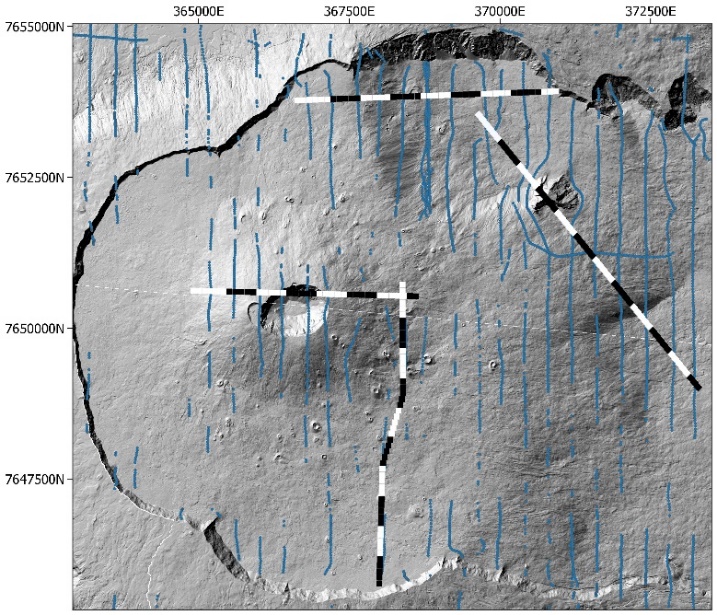  S2c - Location of the 3 655 inverted soundings (blue dots) after the processing scheme adapted to highly resistive setting. | 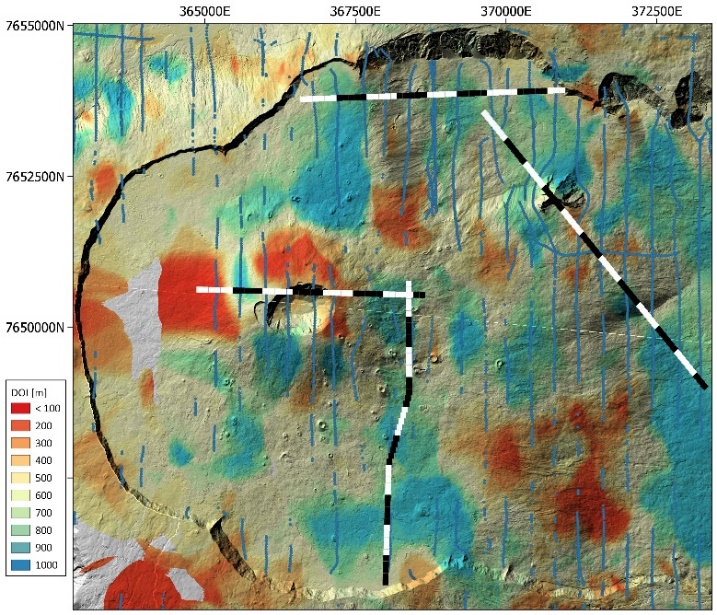  S2d - Depth of Investigation map (DOI) of the 3D resistivity model. |
| 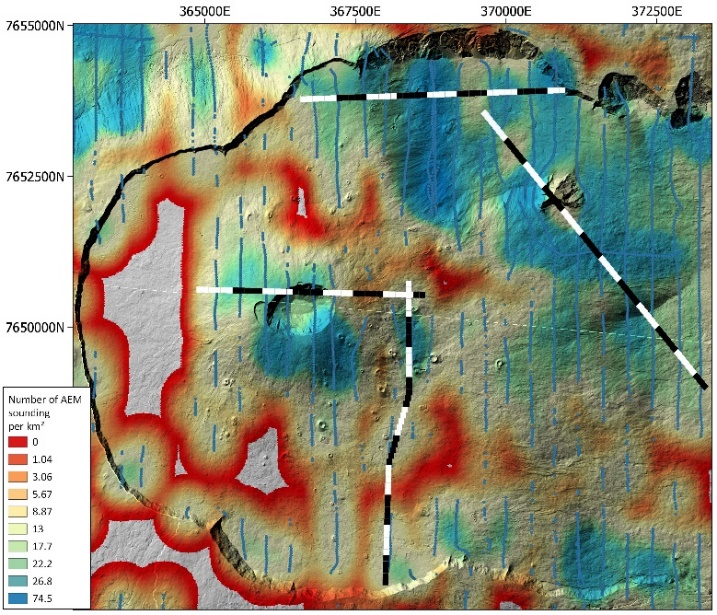 S2e - Number of inverted AEM soundings per square km. | In the five maps, the four 2D resistivity profiles shown in the study are marked by white/black lines (color changes each 500 m). |

**Supplementary – S4:** 2D resistivity maps extracted from the 3D resistivity model

| 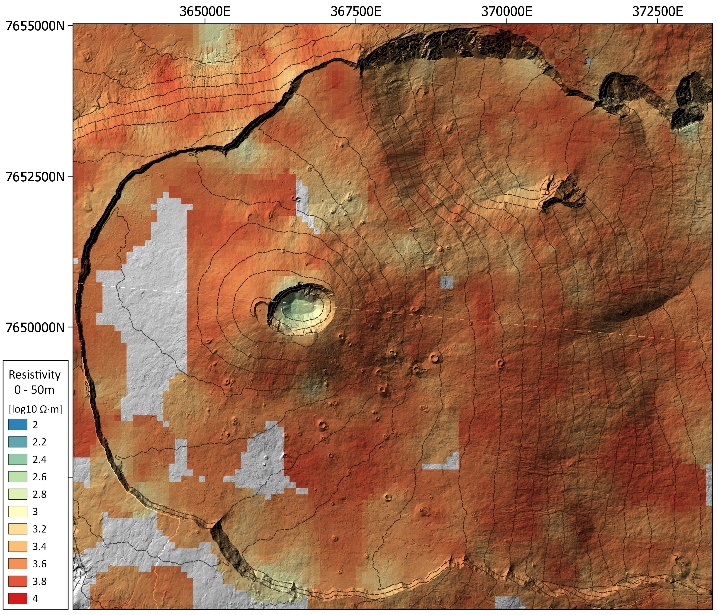  S4a – Resistivity map between 0 and 50 meters depth. | 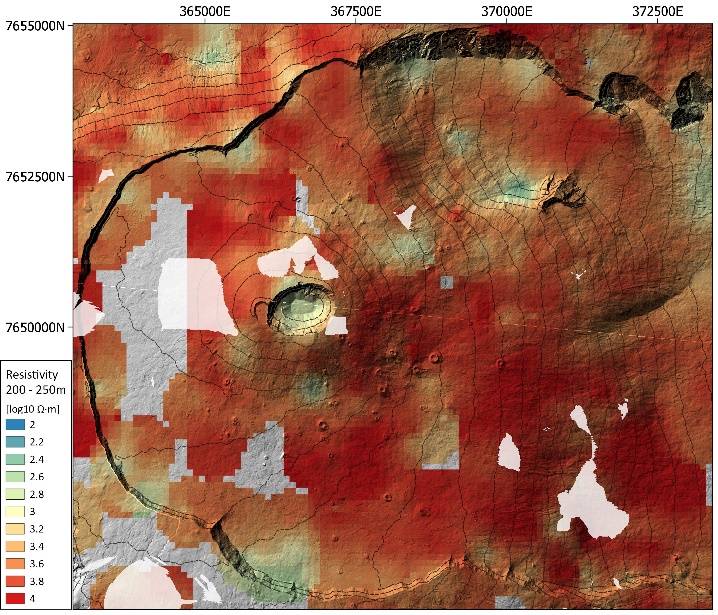  S4b – Resistivity map between 200 and 250 meters depth. |
| --- | --- |
| 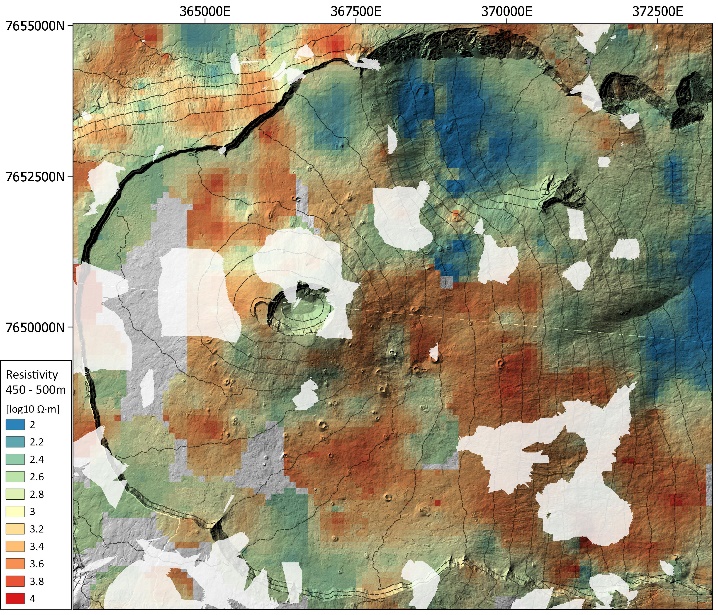  S4c – Resistivity map between 450 and 500 meters depth | 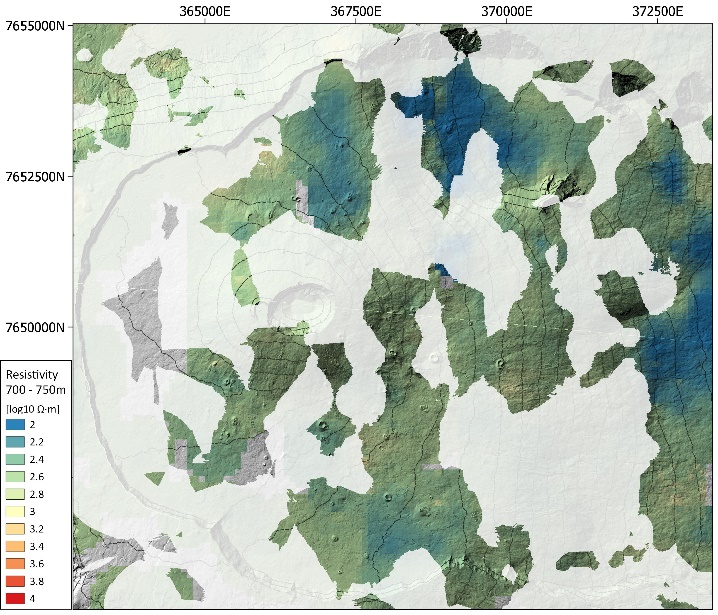  S4d – Resistivity map between 700 and 750 meters depth |
| 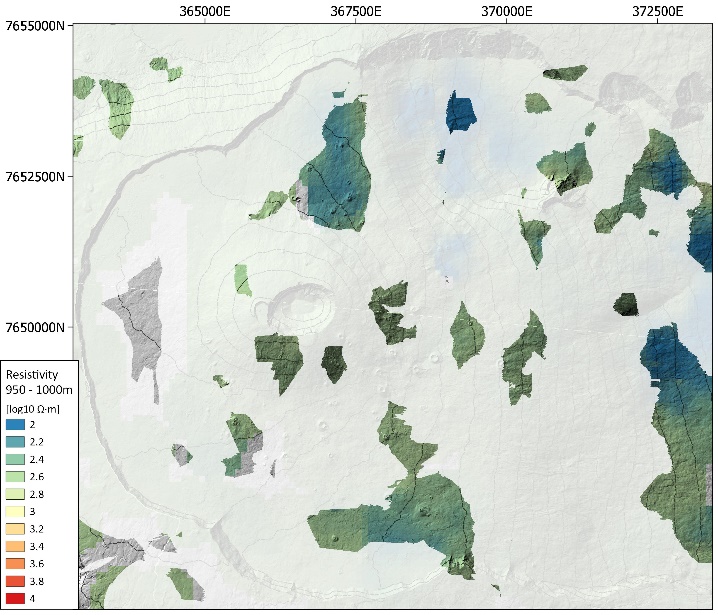 S4e – Resistivity map between 950 and 1000 meters depth. | In the five maps, the resistivity is blanked in function of the sensitivity (DOI) of the resistivity model. |

.
